# Effects of Dietary Supplementation with *Clostridium butyricum* on Growth Performance, Serum Immunity, Intestinal Morphology, and Microbiota as an Antibiotic Alternative in Weaned Piglets

**DOI:** 10.3390/ani10122287

**Published:** 2020-12-03

**Authors:** Yunsheng Han, Chaohua Tang, Ying Li, Yanan Yu, Tengfei Zhan, Qingyu Zhao, Junmin Zhang

**Affiliations:** 1 State Key Laboratory of Animal Nutrition, Institute of Animal Sciences of Chinese Academy of Agricultural Sciences, No. 2 Yuan Ming Yuan West Road, Beijing 100193, China; hanysh17@lzu.edu.cn (Y.H.); jxtangchaohua@sina.com (C.T.); liying@cahg.com.cn (Y.L.); yuyananBNU@163.com (Y.Y.); zhantf2019@sina.com (T.Z.); 2 Scientific Observing and Experiment Station of Animal Genetic Resources and Nutrition in North China of Ministry of Agriculture and Rural Affairs, Institute of Animal Science of Chinese Academy of Agricultural Sciences, Beijing 100193, China; 3 State Key Laboratory of Grassland Agro-Ecosystems, Key Laboratory of Grassland Livestock Industry Innovation, Ministry of Agriculture and Rural Affairs, College of Pastoral Agriculture Science and Technology, Lanzhou University, No. 222 Tian Shui Road, Lanzhou 730020, China

**Keywords:** apparent total tract digestibility, average fecal score, intestinal health, serum antioxidant, short-chain fatty acids, weaning stress

## Abstract

**Simple Summary:**

Many countries have banned the use of antibiotic growth promoters (AGPs), which negatively affect weanling piglets’ growth performance and health. Therefore, it would be valuable to find eco-friendly and non-antibiotic alternatives to AGPs and to evaluate their effects. However, limited information is available on *Clostridium butyricum* applied in weanling piglets. In this study, the results showed that *Clostridium butyricum* administration have positive effects on growth performance, immunity, intestinal morphology, and microbial balance. In conclusion, *Clostridium butyricum* can be used as a potential alternative to AGPs in weanling piglets.

**Abstract:**

This study investigated the effects of *Clostridium butyricum* (*C. butyricum*) use on growth performance, serum immunity, intestinal morphology, and microbiota as an antibiotic alternative in weaned piglets. Over the course of 28 days, 120 piglets were allocated to four treatments with six replicates of five piglets each. The treatments were: CON (basal diet); AGP (basal diet supplemented with 0.075 g/kg chlortetracycline, 0.055 g/kg kitasamycin, and 0.01 g/kg virginiamycin); CBN (basal diet supplemented with normal dosage of 2.5 × 10^8^ CFU/kg *C. butyricum*); and CBH (basal diet supplemented with high dosage of 2.5 × 10^9^ CFU/kg *C. butyricum*). Body weight (BW) and feed consumption were recorded at the beginning and on days 14 and 28 of the experiment, and representative feed samples and fresh feces were collected from each pen between days 26 and 28. Average fecal score of diarrhea was visually assessed each morning during the experimental period. On the morning of days 14 and 28, blood samples were collected to prepare serum for immune and antioxidant parameters measurement. One male piglet close to the average group BW was selected from each replicate and was slaughtered on day 21 of the experiment. Intestinal crypt villi, and colonic microbiota and its metabolites short-chain fatty acids were measured. Compared to the CON group, the CBN and AGP groups significantly decreased (*p* < 0.05) the ratio of feed to weight gain by 8.86% and 8.37% between days 1 and 14, 3.96% and 13.36% between days 15 and 28, 5.47% and 11.44% between days 1 and 28. Dietary treatment with *C. butyricum* and AGPs significantly decreased the average fecal score during the experimental period (*p* < 0.05). The apparent total tract digestibility of dry matter, organic matter, and total carbohydrates in the CBH group were higher respectively at 3.27%, 2.90%, and 2.97%, than those in the CON or AGP groups (*p* < 0.05). Compared to the CON group, the CBH group significantly increased short-chain fatty acids in colon and villus height in the jejunum (*p* < 0.05). The CBN group had higher serum levels of immunoglobulins, interleukin 2 (IL-2), and glutathione peroxidase (GSH-PX) activity, but lower serum levels of IL-1β and IL-6, and a lower aspartate aminotransferase (AST), alkaline phosphatase (ALP), and gamma-glutamyl transpeptidase (γ-GT) activity (*p* < 0.05), while compared to the CON group. Dietary treatment with *C. butyricum* significantly increased the relative abundance of *Streptococcus* and *Bifidobacterium* (*p* < 0.05). In summary, diet with *C. butyricum* increased the growth performance and benefited the health of weaned piglets.

## 1. Introduction

Weaning stress can cause dynamic dysbiosis of the microbiota, dyspepsia, retardation of performance, and even death in piglets [1,2], which can result in huge economic losses in livestock production. As an alternative, antibiotic growth promoters (AGPs) have been used as feed additives to prevent weaning-associated disorders, including an increase in harmful microbial activity and post-weaning diarrhea in weaned piglets [3,4]. However, indiscriminate use of AGPs in animal feed poses a threat to human health related to increased antimicrobial resistance in pathogenic and commensal bacteria and residues in animal products and environment. As a result, the European Union has prohibited the use of AGPs in animal feed since January 2006. In July 2019, Proclamation No. 194 was issued by the Ministry of Agriculture and Rural Villages of the People’s Republic of China aiming to remove AGP, except in traditional Chinese medicine, beginning January, 2020. Given the trend of banning the use of AGPs as feed additives, it is of high priority to find eco-friendly and effective alternatives to AGPs to compensate for the resulting decrease in animal growth performance.

*C. butyricum* is a butyric acid-producing Gram-positive anaerobic and spore-forming bacillus with high tolerance to gastrointestinal environment and is isolated from the intestines of healthy humans and animals [5]. This key characteristic ensures that it arrives to the hind gut and plays an important role in regulating intestinal ecological balance. Previous study in Kunming strain mice has indicated that *C. butyricum* HM754264 by oral gavage at a dosage of 10^6^ CFU/day can obviously promote the proliferation of beneficial bacteria, such as *Lactobacillus* spp. and *Bifidobacterium* spp. [6]. In a germ-free mice study, *C. butyricum* MIYAIRI 588 protected against *Escherichia coli* O157:H7 infection by decreasing its counts, while mice survival rate was increased by 50% [7]. Meanwhile, *C. butyricum* MIYAIRI 588 were capable of inhibiting germination of much larger quantities of *Clostridium difficile* and toxin protein production in vitro [8]. Moreover, in a 28 days feeding experiment, dietary supplementation with *C. butyricum* (Strain No. 1.336) in the quantity of spore state of 1 × 10^8^ CFU/g increased the average daily gain of crossbred piglets (Duroc × Landrace × Large White) [5]. The dosage of *C. butyricum* MIYAIRI 588 containing 10^7^ CFU/g of viable spores decreased antibiotic-associated diarrhea, approximately by 9% [9]. This positive effect may be connected to butyrate, the main metabolite of *C. butyricum*, which has been shown to control weanling diarrhea [10]. However, there is insufficient research evidence and inconsistent results to support the use of *C. butyricum* in swine production. In addition, *C. butyricum* appears to carry a risk of bidirectional regulation [11].

The present study evaluated the effects of dietary supplementation with *C. butyricum* on growth performance, serum immunity, intestinal morphology, and microbiota as a replacement for AGP in weaned piglets. The study aimed to provide a theoretical basis for the application of *C. butyricum* instead of antibiotics in livestock production.

## 2. Materials and Methods

The study was conducted in January, 2019 at the Inner Mongolia Xingdaye Agriculture and Animal Husbandry Company Limited (N 40°10′, E 110°26′), Wulanchabu city, Inner Mongolia province, China. All procedures in this experiment were performed in accordance with the recommendations of “Guidelines on Welfare and Ethical Review for Laboratory Animals” [12] approved by the Institutional Animal Care and Use Committee of the Institute of Animal Sciences of the Chinese Academy of Agricultural Sciences.

### 2.1. Clostridium Butyricum and Antibiotics

*C. butyricum* (China Center for Type Culture Collection CCTCC M 2011384) was obtained by the Academy of Baolai-Leelai Biology Technology (Tai’an, China). The carrier for *C. butyricum* is cornmeal. An antibiotic premix (75 g chlortetracycline, 55 g kitasamycin, and 10 g virginiamycin) was provided by the Inner Mongolia Xingdaye Agriculture and Animal Husbandry Company Limited (Wulanchabu, China).

### 2.2. Weanling Piglets, Diets, and Experimental Design

A total of 120 (48 female and 72 male) crossbred piglets, (Duroc × (Landrace × Yorkshire)), 8.09 ± 0.01 kg of body weight (BW), were weaned at 28 days and used in the experiments. Piglets were randomly allocated to four dietary treatments (six replicates per treatment, with three male and two female piglets per replicate) on the basis of initial BW. The experiment lasted 28 days. A non-medicated corn-soybean basal diet was formulated to meet the National Research Council [13] nutrient requirements for 11–20-kg pigs (Table 1). The dietary treatments included a basal (CON), AGP (basal diet supplemented with 0.075 g/kg chlortetracycline, 0.055 g/kg kitasamycin, and 0.01 g/kg virginiamycin), CBN (basal diet supplemented with 2.5 × 10^8^ CFU/kg *C. butyricum*) [14], and CBH (basal diet supplemented with 2.5 × 10^9^ CFU/kg *C. butyricum*) diets. All piglets were housed in an environmentally controlled room with a hard plastic fully-slotted floor. The adjacent pens were separated by a closed baffle. The room temperature was controlled at 30 °C throughout the first 2 weeks, and then gradually decreased and kept at 26 °C until the end of the experiment.

### 2.3. Sample Collection and Measurements

#### 2.3.1. Growth Performance

Taking each repeat as one unit, BW was recorded at the beginning of each experiment and on days 14 and 28. Feed consumption was recorded on days 14 and 28 to calculate the average daily gain (ADG), average daily feed intake (ADFI), and the ratio of feed to weight gain (F/G).

Clinical signs of diarrhea were visually assessed each morning by observers blinded to the treatments using a five-grade scoring system [15]: 1 = well-formed feces, 2 = slightly soft feces, 3 = soft and partially formed feces, 4 = loose and semiliquid feces, and 5 = watery feces. Then the daily average fecal score per replicate was calculated. Average fecal score per replicate from days 1–14, 15–28, and 1–28 were calculated and used for further statistical analysis.

#### 2.3.2. Apparent Nutrient Digestibility

Representative feed samples of 1.0 kg were collected at the end of the experiment. Between days 26 and 28, approximately 200 g of fresh feces were collected from each pen and immediately frozen at −20 °C. Each three-day collection of feces was pooled by pen, dried at 65 °C for 72 h, and subsequently ground to pass through a 1-mm sieve until further analysis. Crude protein (CP), ether extract (EE), ash, and dry matter (DM) of feed and fecal samples were measured according to the AOAC [16] methods. Diet and feces chromium concentrations were determined by an automatic absorption spectrophotometer (Z-5000; Hitachi, Tokyo, Japan) based on the procedures described by previous study [17]. In addition, total carbohydrates and organic matter (OM) were calculated using the following equation: total carbohydrates = DM − CP − EE − ash, OM = 1 − ash [18]. Apparent total tract digestibility_nutrient_ (ATTD) = 1 − (Cr_diet_ × Nutrient_feces_)/(Cr_feces_ × Nutrient_diet_).

#### 2.3.3. SCFAs Concentration in Colon

After fasting overnight on day 21, one male piglet close to the average group BW was selected from each replicate and was slaughtered 5 min after injecting the anesthetic. Colonic digesta samples were collected from slaughtered piglets. Short chain fatty acids (SCFAs), including acetate, propionate, butyrate, valerate, isobutyrate, and isovalerate, were quantified by an external standard method using gas chromatography (GC) as described by previous study [19,20]. Briefly, 1.5-g colonic chyme samples were added to screw-capped tubes with 6 mL of distilled water. After mixing overnight at 4 °C and centrifugation at 1500 r/min for 10 min at 4 °C, 2 mL of supernatant from each sample were transferred to another centrifuge tube, and 400 μL of meta-phosphoric acid (25% *v*/*v*) were added to remove the protein. The samples were then centrifuged at 12,000 r/min for 10 min at 4 °C. The resulting supernatants (1 mL each) were transferred into gas chromatography sample bottles and analyzed using an Agilent 6890N GC (Palo Alto, CA, USA) coupled to a flame-ionization detector with helium used as the carrier gas. An Agilent FFAP column (30 m × 0.53 mm i.d. × 1.00 μm (film thickness)) was installed for analysis, with a constant flow rate of 4.0 mL/min. Splitless injection volume was 0.2 μL of sample. The injector and detector temperature were 220 °C and 240 °C, respectively. The GC oven temperature was held at 90 °C for 1 min and then increased to 190 °C at a rate of 20 °C/min and held for 3 min. Samples were run in triplicate, with a coefficient of variation less than 15% within triplicate samples used for quality control [19,20].

#### 2.3.4. Immunity and Antioxidant Indices in Serum

On the morning of days 14 and 28, 5-mL blood samples were collected from one male piglet from each replicate via jugular vein puncture into a vacutainer. After 2 h, blood samples were centrifuged at 3000× *g* at 4 °C for 10 min to recover the serum, which was stored at −20 °C until further analysis. Serum immunoglobulins (IgG, IgA, and IgM), interleukins (IL-1β, IL-2, and IL-6), and tumor necrosis factor (TNF-α) were measured using an ELISA kit (Shanghai Enzyme-linked Biotechnology, Co., Ltd., Shanghai, China). Serum antioxidant indices, including total antioxidant capacity (T, -AOC), glutathione peroxidase (GSH-PX), inhibiting ability of hydroxyl radical (OH), superoxide dismutase (SOD), hydrogen peroxide (H_2_O_2_), nitric oxide (NO), and Cholinesterase (CHE) were measured using biochemical methods following manufacturer instructions provided for each reagent kit (Nanjing Jiancheng Bioengineering Institute, Nanjing, China). Serum alanine transaminase (ALT), aspartate aminotransferase (AST), alkaline phosphatase (ALP), and gamma-glutamyl transpeptidase (γ-GT) were measured with an automatic biochemical analyzer (KHB-1280, Shanghai, China).

#### 2.3.5. Intestinal Morphology

After slaughtering piglets, the intestines were divided into three parts, including duodenum, jejunum, and ileum, according to the method described by previous study [21]. Then, the middle of duodenum, jejunum, and ileum with 2 cm long intestinal canal was respectively collected, slightly washed with saline, and immediately fixed in 4% formalin, stored at 4 °C. The intestinal specimens were embedded in paraffin, sectioned (4 μm), and stained with hematoxylin, eosin, and periodic acid-Schiff. Three intact crypt villi in each sample were selected, and villus height and adjoining crypt depth were determined using an image analysis system (Version 1, Leica Imaging Systems Ltd., Cambridge, UK) [22]. Villus height was measured from the apex to the base of the villus crypt junction along its axis, and crypt depth was defined as the depth of the invagination between adjacent villi [23]. The ratio of villus height to crypt depth was calculated.

#### 2.3.6. Microbiota in Colon

Colonic digesta samples were collected from slaughtered piglets and immediately stored at −80 °C. Bacterial genomic DNA was extracted from each sample (Qiagen DNA Stool Mini Kit, Duesseldorf, Germany). DNA was quantified with a NanoDrop 2000 spectrophotometer (Thermo Scientific, Wilmington, NC, USA) and further assessed by running on 1% agarose gels. The V3–V4 hypervariable region of 16S rRNA genes was amplified using specific primer pairs (forward 5′-ACTCCTACGGGAGGCAGCA-3′ and reverse 5′-GGACTACHVGGGTWTCTAAT-3′) with barcodes to construct the sequencing libraries (TruSeq^®^ DNA PCR-Free Sample Prep Kit, Illumina, San Diego, CA, USA). The qualified DNA libraries were loaded in a NovaSeq platform with 2 × 250 bp paired-end sequencing. The paired-end reads were obtained and merged using FLASH software (V1.2.7, http://ccb.jhu.edu/software/FLASH/). Operational taxonomic units (OTUs) with a 97% identity were gathered with Uparse (ver. 7.1, http://drive5.com/uparse/). Taxonomic annotation was performed using the Mothur algorithm (70% confidence) with the Silva Database (http://www.arb-silva.de/). Taxonomic composition of the bacterial community was then analyzed.

### 2.4. Statistical Analysis

Data were analyzed using one-way analysis of variance according to the comparative average and general line model procedures in SPSS 22.0 (IBM Corp., Armonk, NY, USA). The differences among treatment means for the growth performance, serum indices, ATTD, SCFAs concentration, and relative abundance of microbiota were analyzed using Duncan’s multiple-range test and least significant difference (LSD) post hoc tests. *p*-values < 0.05 were considered significant.

## 3. Results

### 3.1. Growth Performance

Compared to the CON group, dietary supplementation with *C. butyricum* (2.5 × 10^8^ CFU/kg) increased the feed efficiency by significantly decreasing F/G (*p* < 0.05) and decreased average fecal score during days 1−14, 15−28, and 1−28 (*p* < 0.05, Table 2). Supplementation with *C. butyricum* (2.5 × 10^9^ CFU/kg) and AGPs significantly decreased the F/G during days 1−14 (*p* < 0.05), and dietary with AGPs significantly decreased average fecal score during days 1−14, 15−28, and 1−28 (*p* < 0.05) compared to the CON group.

### 3.2. Apparent Nutrient Digestibility

The CBH group significantly increased ATTD of DM, OM, and total carbohydrates compared to the CON group (*p* < 0.05) and significantly increased ATTD of OM and total carbohydrates in comparison to the CBN group (*p* < 0.05, Table 3). While ATTD of EE in the CBN group increased by approximately 7.00% compared to the CON and AGP groups, no significant differences were found between the CBN and CON, AGP, or CBH groups (*p* > 0.05).

### 3.3. SCFAs Concentration in Colon

Compared to CON group, the CBH group significantly increased the concentration of acetic, propionic, and butyric acids and total SCFAs in colon (*p* < 0.05, Table 4). While the CBN group significantly increased the acetic acid, the isobutyric, valeric, isovaleric acids were decreased (*p* < 0.05). Concentrations of propionic, butyric, isobutyric, valeric, and isobaleric acids in the CBN group were lower than those in the CBH group (*p* < 0.05). However, there were no significant differences in total SCFAs between the CBN and CBH groups (*p* > 0.05).

### 3.4. Immunity and Antioxidant Indices in Serum

On day 14, the CBN group exhibited a higher IgM serum level, inhibiting hydroxyl radical ability and GSH-PX activity trend (*p* = 0.066), while the AGP group showed a lower TNFα serum level in comparison to the CON group (*p* < 0.05, Table 5). On day 28, compared to the CON group, the CBN group significantly increased serum levels of IgA, IgM, IgG, and IL-2 and GSH-PX activity, inhibited hydroxyl radical ability, and decreased IL-1β and IL-6 serum levels (*p* < 0.05). Serum IgG level and hydroxyl radical inhibition ability in the CBN group was higher, while concentration of NO was lower than that in the AGP group (*p* < 0.05). The CBH group also modestly increased serum levels of immunoglobulins (Igs) and decreased the levels of pro-inflammatory factors, including IL-1β, IL-6, and TNF α, on day 28 compared to the CON group (*p* > 0.05). Piglets in the AGP, CBN, and CBH groups had lower ALP, γ-GT, and CHE activities compared with piglets in the CON group (*p* < 0.05) on day 14. In addition, γ-GT and CHE in the CBN and CBH groups were lower than those in the AGP group (*p* < 0.05). On day 28, AST and γ-GT activity in the CON, AGP, and CBN groups were lower than those in the CBH group (*p* < 0.05), and AST activity in the CBN group was lower than that in the CON and AGP groups (*p* < 0.05). Piglets in the CON and CBN groups had lower CHE activity than piglets in the AGP and CBH groups (*p* < 0.05).

### 3.5. Intestinal Morphology

Dietary treatment with the CBH diet significantly increased villus height and crypt depth of the jejunum compared to the CON group (*p* < 0.05, Table 6). Villus height of the jejunum and ileum in the CBN group was slightly higher than that in the CON group, with no differences present between the CBN and CBH groups (*p* > 0.05).

### 3.6. Microbiota in Colon

The dominant phyla included *Firmicutes, Proteobacteria*, and *Bacteroidetes*, which represented more than 57.15, 6.56, and 14.73% of the total community, respectively (Table 7). Compared to the CON group, the relative abundance of *Firmicutes* and *Proteobacteria* in the CBN group was decreased by approximately 10.24 and 54.78%, respectively (*p* > 0.05), while the relative abundance of *Bacteroidetes* was increased by 31.70% (*p* > 0.05). Of the 50 most dominant genera (18 genera were listed), dietary treatment with *C. butyricum* significantly increased the relative abundance of *Streptococcus* and *Bifidobacterium* (*p* < 0.05) and slightly increased the relative abundance of *Butyricicoccus* (*p* > 0.05). The relative abundance of *Sarcina*, *Blautia*, and *Actinobacillus* (*p* > 0.05) was decreased compared to that in the CON and AGP groups.

## 4. Discussion

*C. butyricum* has been studied as a substitute for antibiotics in recent years, with most research concentrating on its effects on broilers. These studies have shown that *C. butyricum* can promote growth performance and improve nutrient utilization efficiency in broilers [24,25]. However, few studies indicated that *C. butyricum* did not affect growth performance in broilers and piglets [26,27]. Limited studies in piglets have shown that *C. butyricum* supplementation significantly decreased F/G and average fecal score, having had no effects on those indices with the same strain and dose in another experiment [28]. A plausible reason for this observation might be associated with diet type, since *C. butyricum* with a relatively less digestible diet showed positive effects. The present study demonstrated that *C. butyricum* supplementation significantly increased feed efficiency and average fecal score in weaned piglets, which was as effective as AGPs. This is consistent with the previous findings, which showed that dietary *C. butyricum* increased growth performance and enhanced feed efficiency with a significant decrease of F/G ratio, and diarrhea rate in weaned piglets [5,29]. Thus, it can be hypothesized that improved feed efficiency in piglets fed the *C. butyricum* diet might be related to greater nutrient ATTD and better intestinal health.

Contrary to research on other antibiotic alternatives, only a few studies have measured ATTD in piglets fed probiotics, especially for diets with *C. butyricum*. Previous studies have suggested that probiotic supplementation can improve digestibility of DM, CP, and crude fiber [30,31]. As expected, the present study suggested that supplementation with *C. butyricum* increased ATTD of DM, OM, and total carbohydrates compared to the CON group. This indicated that dietary *C. butyricum* increased the utilization efficiency of nutrients, which accounts for the enhanced feed efficiency of piglets. A probable mechanism by which *C. butyricum* increases ATTD may be associated with a healthy intestinal environment in response to increased digestive enzyme activity and large amounts of SCFAs [25]. A previous report indicated that administration of *C. butyricum* to Rex rabbits enhanced the digestive enzyme activity, including trypsin, α-amylase, chymotrypsin, and cellulase activity to varying degrees [24]. Mounting evidence suggests that dietary supplementation with *C. butyricum* increases the concentrations of acetic acid, butyric acid, and total SCFAs in the intestinal digesta [11,26,29,32,33], which reduce gut pH. These reports are basically consistent with the present study. A lower gut pH has been reported to contribute to nutrient digestibility due to their antibacterial effects on enterobacteria and increased secretion of digestive enzymes [34,35]. In addition, SCFAs benefit animals by providing energy for intestinal mucosal epithelial cells.

Intestinal morphology is commonly considered an important mechanical defense of barrier functions, where villus height and crypt depth are used for evaluating intestinal development and absorptive function [36]. A previous study has shown that dietary treatment with *C. butyricum* had a higher ileac villous height and lower crypt depth in broiler chickens [37] and broiler chickens challenged with *E. coli* K88 [38]. In a piglet study, piglets administered *C. butyricum* significantly increased jejunal villus height and ratio of the jejunal height to crypt depth [29]. The present study further demonstrated that dietary treatment with *C. butyricum* increased the intestinal villus height and crypt depth. The underlying mechanism was associated with metabolites of microbiota, especially butyric acid, which acts as a functional low molecular weight substance. Butyric acid was generally the main source of energy for epithelial cells and activated the mitotic activity of crypt enteric cells [39], potentially resulting in longer villi. Previous studies have demonstrated an increase in intestinal villus height after butyrate supplementation in weaning piglets [35,40]. Thus, these reports have indicated that *C. butyricum* metabolites may play an important role in improving gut health and increasing nutrient utilization.

Serum Ig concentration can be used as a key parameter to reflect animal humoral immune status. Relatively high levels seem to be particularly necessary for weanling piglets because they are susceptible to stressors and pathogens [41]. In the present study, dietary treatment with *C. butyricum* significantly increased serum levels of IgA, IgM, and IgG compared to the CON diet. This was consistent with previous studies. Diet supplemented with *C. butyricum* has increased the level of serum IgM in piglets on day 14, and the level of serum IgG, IgA, and IgM in piglets on day 28 [29]. It has been reported that dietary supplementation with *C. butyricum* stimulated secretion of Igs in broilers [24,42], mice [43], and Peyer’s patch cell [44]. Interestingly, SCFAs produced by *C. butyricum* can improve the immune function of mice by enhancing B cell antibody production [45]. These results demonstrated that *C. butyricum* plays a positive role in enhancing lymphocyte response and immune status, contributing to animal protection against pathogenic microorganisms. Cytokines, including IL-1β, TNF-α, and IL-6 are key pro-inflammatory events that induce an inflammatory response in mammals. IL-1β usually increases the margination of lymphocytes by decreasing local blood flow rate, contributing to pathogen exclusion [46]. IL-6 acts on the mesenchymal cells to induce recruitment of macrophages and polymorphonuclear leukocytes to eliminate pathogens [47]. Previous report indicated that dietary supplementation with *C. butyricum* significantly decreased production of IL-1β, IL-8, and TNF-α to a different extent in livers, spleens, and cecal tissues of chicken challenged with *Salmonella enteritidis* [48]. This effect was consistent with the present results. A similar result was found in a weaning Rex rabbit study where treatment with *C. butyricum* decreased the ileum levels of IL-6 and TNF-α [49]. A potential mechanism responsible for this action might be associated with the Toll-like receptor (TLR)-mediated nuclear factor-kappa enhancer binding protein (NF-κB) signaling pathway. *C. butyricum* can also mitigate inflammation in chickens after being challenged with *Salmonella enteritidis* by down-regulating the TLR4- and NF-κB-dependent pathway [48]. Furthermore, the present study indicated that a diet with *C. butyricum* significantly increased serum IL-2 level compared to the CON group. IL-2 is a pleiotropic cytokine that is required to prevent chronic inflammation and is necessary to maintain regulatory T cell secretion of IL-10 recognized as an anti-inflammatory cytokine [47,50]. These reports have demonstrated that *C. butyricum* might be a key modulator needed to maintain immunological host homeostasis.

Reactive oxygen species (ROS) have been associated with host defense, inflammation, and tissue damage [51]. Antioxidant enzymes participate in inactivation of ROS and degradation of superoxide anions and hydrogen peroxide [52]. In the current study, the CBN group significantly increased the inhibition ability of hydroxyl radical and GSH-PX activity and modestly decreased H_2_O_2_ serum concentration. This was in agreement with previous studies [24,49], which showed that diets with *C. butyricum* can increase the activity of GSH-PX, SOD, and CAT in intestine of broiler and Rex rabbits. In addition, *C. butyricum’s* main metabolite butyric acid can modulate oxidative damage by reducing ROS and increasing the levels of antioxidative enzymes [53,54]. Improved immune function might partially contribute to enhance the antioxidant ability and suppress ROS production, because ROS are considered indispensable for host defense against an inflammatory threat [51]. ALT, AST, ALP, γ-GT, and CHE play an important role in liver function as main indicators. A previous study revealed that *C. butyricum* supplementation resulted in a considerable reduction in the serum ALT and AST activities in piglets [29]. In the mice model, *C. butyricum* also relieved acute liver damage induced by carbon tetrachloride [55]. Our results indicated that diet supplemented with *C. butyricum* decreased AST, ALP, γ-GT, and CHE activies, particularly in the CBN group.

The above positive effects of dietary *C. butyricum* may be mediated by improvements in microbiota composition. Phyla of *Firmicutes* and *Bacteroidetes* are the dominant members of colonic microbiota and most *Bacteroide* strains are amylolytic, hemicellulolytic, or proteolytic bacteria [56]. *Bacteroides* identified in the cecal samples contribute 31.9% of glycoside hydrolase genes, 26.1% of polysaccharide lyase genes, 20.7% of carbohydrate esterase genes, and 16.2% of glycosyltransferase genes [57]. In contrast, *Proteobacteria* are facultative anaerobic bacteria that do not specialize in fiber consumption and might even interfere with host nutrition by consuming fermentation products of carbon dioxide when oxygen is present [58]. The present study indicated that a modest increase in relative abundance of *Bacteroidetes* in the *C. butyricum* treatment might have been responsible for the depletion of *Proteobacteria* and *Firmicute* representatives. This was consistent with previous study [59]. Moreover, *Bacteroides* can hydrolyze indigestible dietary polysaccharides and supply 10–15% of daily calories as fermentation products [60]. These might be some of the reasons that diets with *C. butyricum* had a lower F/G and higher ATTD compared to the CON group in the present study.

Actinobacteria are associated with maintenance of intestinal mucosal surface homeostasis and acetate production, in which *Bifidobacterium* as an obligate anaerobic bacteria is conducive to the development of immune system [61]. *Sarcina* is a genus of Gram-positive obligate anaerobic tetrad cluster cocci bacteria that have been frequently recovered from gastric samples of patients suffering from gastroparesis and peritonitis [62]. As a result of the present study, the CBN diet treatment significantly increased the relative abundance of *Bifidobacterium* compared to other treatments, while the relative abundance of *Sarcina* in the CBN, CBH, and AGP groups was modestly decreased. The present study indicated that supplementation with *C. butyricum* enriched the beneficial bacteria and decreased potential pathogens. Moreover, there seems to be a close correlation between microbiota and immunity or antioxidant resistance. It has been reported that *Bifidobacterium* can suppress pro-inflammatory cytokines by inhibiting NF-κB activation [63,64]. Correlation analysis showed that the relative abundance of *Streptococcus* and *Roseburia* was positively correlated with serum activity of T-AOC and GSH-PX and SCFAs concentrations in the intestine, while relative abundance of *Blautia*, *Parabacteroides*, and *Marvinbryantia* was negatively correlated with that of T-AOC, GSH-PX, and SCFAs [65]. This was consistent with the present results. Increased SCFAs levels resulting from dietary supplementation with *C. butyricum* in the present study might be associated with a significant increase in *Streptococcus* and *Bifidobacterium* and a modest increase in *Bacteroides* and *Anaerostipes*. These can directly produce SCFAs, including acetic and butyric acids, or indirectly convert lactate to acetate via the methylmalonyl-CoA pathway, and finally utilize both acetate and lactate to synthesize butyrate via the butyryl-CoA:acetate-CoA-transferase route [5,66]. These results indicated that *C. butyricum* might shape a better metabolic milieu for the intestinal ecosystem by regulating microbiota and its metabolites of SCFAs, which benefit ATTD, antioxidant resistance, and immune function.

Compared to diet intervention and host genetics, weaning age might be an ignored factor that can generate collateral effects influencing gut development, and may result in microbial dysbiosis, subsequent diarrhea, and growth check [67]. Longitudinal studies of infants have demonstrated that adult-associated microbial community begin to dominate the infant microbiota post-weaning [68], indicating early weaning prematurely displaced beneficial infant-associated microbes, and then negative affected gut health [69]. With the development of livestock production, early weaning may become common, so that the weaning age of piglet decreased from 28 to 21 days old. Thus, the early weaning-associated problems obviously become prominent. Early colonization of beneficial microbe may be an effective way to positively impact animal gut and lifelong health. As reported, diet supplemented with *C. butyricum* reshaped microbial community structure, and enhanced the intestinal health and nutrient absorption in weaned piglets by improving intestinal histology [27,29]. Butyrate as the main metabolite of *C. butyricum* could directly provide energy for enterocytes to maintain the mucosal barrier [69]. Moreover, the microbial diversity significantly increased with the age of pig [70], indicating the piglet has a lower microbial diversity when suffering early weaning. Therefore, diet supplemented with *C. butyricum* may have a higher probability of *C. butyricum* colonization, and then contribute more to the gut and host health at 21 days weaning, compared to 28 days weaning. However, more research is needed to better evaluate the effects of *C. butyricum* in different weaning time.

## 5. Conclusions

The study results suggest that dietary treatment with *C. butyricum* can increase the growth performance, decrease average fecal score, protect liver, and enhance health status in weaned piglets by improving serum immune function, antioxidant ability, and regulating the gut microbiota. *C. butyricum* could serve as a potential alternative to AGP administration in weanling piglets to promote growth and improve gut health. Moreover, it might present a better effect when applying in early weaning or even pre-weaning.

## Figures and Tables

**Table 1:** Ingredients and chemical composition of experimental diets (as-fed basis).

Ingredient, %	Content	Nutrient Composition ^2^, %	
Extruded corn	55.00	Digestible Energy, MJ kg^−1^	14.50
Soybean meal	11.30	Crude protein	19.10
Extruded soybean	10.00	Calcium	0.82
Fish meal	5.00	Total phosphorus	0.72
Soybean protein concentrate	4.00	Digestible phosphorus	0.49
Whey powder	8.00	Digestible lysine	1.23
Sucrose	2.00	Digestible methionine	0.36
Soy oil	1.50	Digestible threonine	0.74
Dicalcium phosphate	1.00	Digestible tryptophan	0.20
Limestone	0.50		
Salt	0.20		
Chromium oxide	0.25		
L-Lysine-HCl	0.30		
DL-Methionine	0.20		
L-Threonine	0.15		
L-Tryptophan	0.10		
Vitamin and mineral mixture ^1^	0.50		

^1^ Provided per kg of diet: vitamin A, 2200 IU; vitamin D_3_, 220 IU; vitamin E, 11 IU; vitamin K_3_, 0.5 mg; vitamin B_12_, 0.015 mg; riboflavin, 4 mg; niacin, 30 mg; pantothenic acid, 10 mg; choline chloride, 400 mg; folic acid, 0.3 mg; thiamine, 1.5 mg; vitamin B_6_, 3 mg; biotin, 0.1 mg; zinc, 100 mg; manganese, 4 mg; iron, 84 mg; copper, 6 mg; iodine, 0.14 mg; and selenium, 0.35 mg. ^2^ Nutrient levels are calculated.

**Table 2:** Effect of dietary *C. butyricum* on growth performance and average fecal score in weaned piglets (mean ± SE ^2^).

Item	Dietary Treatments ^1^				*p*-Value
	CON	AGP	CBN	CBH	
Body weight, kg					
Initial	8.09 ± 0.01	8.09 ± 0.01	8.10 ± 0.005	8.09 ± 0.01	0.376
Day 14	10.18 ± 0.24	10.43 ± 0.14	10.24 ± 0.16	10.15 ± 0.10	0.293
Day 28	14.11 ± 0.49	14.57 ± 0.55	14.33 ± 0.17	14.04 ± 0.33	0.416
Days 1–14					
ADFI ^3^, kg	0.325 ± 0.035	0.333 ± 0.016	0.303 ± 0.019	0.294 ± 0.016	0.284
ADG ^3^, kg	0.161 ± 0.019	0.180 ± 0.011	0.164 ± 0.012	0.158 ± 0.007	0.302
F/G ^3^	2.03 ± 0.07 ^a^	1.86 ± 0.05 ^b^	1.85 ± 0.05 ^b^	1.85 ± 0.03 ^b^	0.035
Average fecal score	2.22 ± 0.07 ^a^	1.67 ± 0.12 ^c^	1.87 ± 0.02 ^b,c^	2.02 ± 0.07 ^a,b^	0.001
Days 15–28					
ADFI, kg	0.513 ± 0.036	0.517 ± 0.043	0.466 ± 0.019	0.468 ± 0.015	0.304
ADG, kg	0.256 ± 0.025	0.271 ± 0.033	0.268 ± 0.016	0.246 ± 0.015	0.510
F/G	2.02 ± 0.11 ^a^	1.94 ± 0.09 ^a,b^	1.75 ± 0.06 ^b^	1.92 ± 0.08 ^a,b^	0.051
Average fecal score	2.09 ± 0.15 ^a^	1.69 ± 0.09 ^b^	1.70 ± 0.07 ^b^	1.97 ± 0.09 ^a,b^	0.034
Days 1–28					
ADFI, kg	0.416 ± 0.033	0.422 ± 0.026	0.382 ± 0.011	0.380 ± 0.014	0.254
ADG, kg	0.207 ± 0.018	0.224 ± 0.020	0.214 ± 0.006	0.202 ± 0.011	0.352
F/G	2.01 ± 0.07 ^a^	1.90 ± 0.06 ^a,b^	1.78 ± 0.03 ^b^	1.89 ± 0.05 ^a,b^	0.067
Average fecal score	2.16 ± 0.07 ^a^	1.67 ± 0.10 ^b^	1.79 ± 0.04 ^b^	2.00 ± 0.07 ^a^	0.001

^a–c^ Different superscripts within a row indicate a significant difference (*p* < 0.05). ^1^ Dietary treatments: CON = basal diet; AGP = basal diet supplemented with 0.075 g/kg chlortetracycline, 0.055 g/kg kitasamycin, and 0.01 g/kg virginiamycin; CBN = basal diet supplemented with 2.5 × 10^8^ CFU/kg *C. butyricum*; CBH = basal diet supplemented with 2.5 × 10^9^ CFU/kg *C. butyricum*. ^2^ SE: standard error of the mean. Each treatment group contains six replicates (*n* = 6), and each replicate includes five piglets. ^3^ ADFI: average daily feed intake; ADG: average daily gain; F/G: ratio of feed to weight gain.

**Table 3:** Effect of dietary *C. butyricum* on feed nutrient ATTD (mean ± SE ^2^, %).

Item	Dietary Treatments ^1^				*p*-Value
	CON	AGP	CBN	CBH	
Dry Matter	81.99 ± 0.43 ^a^	81.96 ± 0.72 ^a^	82.90 ± 0.63 ^a,b^	84.67 ± 0.60 ^b^	0.024
Crude Protein	76.42 ± 1.47	75.71 ± 1.61	76.00 ± 1.36	77.44 ± 1.46	0.460
Ether Extract	62.90 ± 1.20	62.51 ± 1.13	67.11 ± 2.75	66.55 ± 1.33	0.128
Organic Matter	83.75 ± 0.50 ^a^	83.43 ± 0.80 ^a^	84.11 ± 0.72 ^a^	86.18 ± 0.58 ^b^	0.044
Total Carbohydrates	88.00 ± 0.35 ^a^	87.72 ± 0.50 ^a^	88.30 ± 0.53 ^a^	90.61 ± 0.17 ^b^	0.001

^a,b^ Different superscripts within a row indicate a significant difference (*p* < 0.05). ^1^ Dietary treatments: CON = basal diet; AGP = basal diet supplemented with 0.075 g/kg chlortetracycline, 0.055 g/kg kitasamycin, and 0.01 g/kg virginiamycin; CBN = basal diet supplemented with 2.5 × 10^8^ CFU/kg *C. butyricum*; CBH = basal diet supplemented with 2.5 × 10^9^ CFU/kg *C. butyricum*. ^2^ SE: standard error of the mean. Each treatment group contains six replicates (*n* = 6), and each replicate is a pen unit.

**Table 4:** Effect of dietary *C. butyricum* on short-chain fatty acids in colonic chyme (mean ± SE ^2^, µmol/g).

Item	Dietary Treatments ^1^				*p*-Value
	CON	AGP	CBN	CBH	
Acetic acid	55.66 ± 5.10 ^a^	60.78 ± 3.38 ^a,b^	68.23 ± 1.70 ^b,c^	71.66 ± 1.57 ^c^	0.008
Propionic acid	19.90 ± 0.80 ^a^	21.37 ± 0.72 ^a^	21.82 ± 1.31^a^	24.90 ± 1.16 ^b^	0.017
Butyric acid	7.87 ± 0.93 ^a^	7.35 ± 0.38 ^a^	7.81 ± 0.61^a^	10.86 ± 1.28 ^b^	0.021
Isobutyric acid	0.90 ± 0.07 ^b^	0.40 ± 0.06 ^a^	0.55 ± 0.07 ^a^	0.80 ± 0.08 ^b^	<0.01
Valeric acid	1.97 ± 0.26 ^b^	0.99 ± 0.09 ^a^	1.16 ± 0.22 ^a^	2.16 ± 0.19 ^b^	<0.01
Isovaleric acid	1.17 ± 0.09 ^b^	0.61 ± 0.05 ^a^	0.75 ± 0.11 ^a^	1.07 ± 0.09 ^b^	<0.01
Total short chain fatty acids	87.47 ± 8.06 ^a^	91.50 ± 4.46 ^a^	100.32 ± 2.40 ^a,b^	111.45 ± 9.43 ^b^	0.006

^a–c^ Different superscripts within a row indicate a significant difference (*p* < 0.05). ^1^ Dietary treatments: CON = basal diet; AGP = basal diet supplemented with 0.075 g/kg chlortetracycline, 0.055 g/kg kitasamycin, and 0.01 g/kg virginiamycin; CBN = basal diet supplemented with 2.5 × 10^8^ CFU/kg *C. butyricum*; CBH = basal diet supplemented with 2.5 × 10^9^ CFU/kg *C. butyricum*. ^2^ SE: standard error of the mean. Each treatment group contains six replicates (*n* = 6), and each replicate includes one piglet.

**Table 5:** Effect of dietary *C. butyricum* on serum immunity and antioxidant ability (mean ± SE ^2^).

Item	Dietary Treatments ^1^				*p*-Value
	CON	AGP	CBN	CBH	
Day 14					
IgA ^3^, µg mL^−1^	75.46 ± 6.19 ^a^	77.76 ± 6.73 ^a,b^	90.16 ± 4.97 ^a,b^	93.33 ± 5.88 ^b^	0.047
IgM ^3^, mg mL^−1^	1.58 ± 0.17 ^a^	2.01 ± 0.18 ^a^	2.60 ± 0.13 ^b^	1.93 ± 0.10 ^a^	0.001
IgG ^3^, mg mL^−1^	5.24 ± 0.19	5.46 ± 0.18	5.70 ± 0.17	5.35 ± 0.23	0.142
IL-1β ^3^, pg mL^−1^	116.73 ± 5.28	103.11 ± 4.22	116.16 ± 2.21	108.74 ± 5.71	0.065
IL-2 ^3^, pg mL^−1^	102.69 ± 2.05	100.14 ± 3.12	97.03 ± 3.09	97.83 ± 2.43	0.190
IL-6 ^3^, pg mL^−1^	275.05 ± 5.74	267.11 ± 8.36	264.31 ± 6.33	273.59 ± 6.98	0.327
TNFα ^3^, pg mL^−1^	58.46 ± 1.74 ^a^	53.10 ± 0.97 ^b^	55.99 ± 1.13 ^a,b^	54.78 ± 2.36 ^a,b^	0.032
GSH-PX ^3^, U mL^−1^	582.48 ± 35.98	667.53 ± 30.20	674.93 ± 24.48	584.00 ± 31.07	0.066
OH ^3^ U mL^−1^	760.05 ± 32.09 ^a^	609.66 ± 17.45 ^b^	815.80 ± 36.27 ^a^	632.87 ± 19.70 ^b^	<0.001
SOD ^3^ U mL^−1^	28.99 ± 3.82	28.42 ± 1.00	21.25 ± 3.90	28.43 ± 2.00	0.103
T-AOC ^3^ mMol L^−1^	0.75 ± 0.02	0.75 ± 0.04	0.74 ± 0.03	0.78 ± 0.01	0.714
H_2_O_2_^3^ mMol L^−1^	45.19 ± 6.82 ^a,b^	35.99 ± 1.38 ^a^	41.45 ± 3.55 ^a,b^	50.52 ± 2.04 ^b^	0.019
NO ^3^ µmol mL^−1^	222.86 ± 22.68	206.19 ± 22.77	202.85 ± 5.26	173.33 ± 5.50	0.064
ALT ^3^ U L^−1^	54.56 ± 1.35	51.91 ± 0.92	51.77 ± 0.93	51.45 ± 1.52	0.112
AST ^3^ U L^−1^	25.92 ± 0.54 ^a,b^	25.89 ± 1.32 ^a,b^	21.89 ± 0.62 ^b^	33.56 ± 1.44 ^a^	<0.001
ALP ^3^ U L^−1^	84.29 ± 0.80 ^a^	62.65 ± 0.71 ^c^	76.61 ± 2.39 ^b^	63.44 ± 1.81 ^c^	<0.001
γ-GT ^3^ U L^−1^	31.60 ± 0.65 ^a^	24.08 ± 0.63 ^b^	20.56 ± 0.64 ^c^	21.48 ± 0.82 ^c^	<0.001
CHE ^3^ U L^−1^	625.83 ± 10.43 ^a^	554.48 ± 13.03 ^b^	447.97 ± 13.21^c^	363.13 ± 16.45 ^d^	<0.001
Day 28					
IgA, µg mL^−1^	92.34 ± 8.03 ^a^	113.07 ± 8.65 ^b^	125.82 ± 3.18 ^b^	119.33 ± 5.45 ^b^	0.012
IgM, mg mL^−1^	2.40 ± 0.15 ^a^	2.77 ± 0.14 ^a,b^	2.87 ± 0.10 ^b^	2.67 ± 0.17 ^a,b^	0.033
IgG, mg mL^−1^	6.01 ± 0.26 ^a^	6.52 ± 0.23 ^a^	7.44 ± 0.17 ^b^	6.42 ± 0.13 ^a^	0.001
IL-1β, pg mL^−1^	101.28 ± 4.05 ^a^	80.58 ± 5.65 ^c^	87.70 ± 3.16 ^b,c^	97.47 ± 3.95 ^a,b^	0.011
IL-2, pg mL^−1^	103.53 ± 3.02 ^a^	110.89 ± 3.76 ^a,b^	118.77 ± 3.79 ^b^	111.46 ± 2.82 ^a,b^	0.038
IL-6, pg mL^−1^	287.41 ± 5.81 ^a^	224.87 ± 6.87 ^b^	240.04 ± 5.04 ^b^	246.91 ± 10.15 ^b^	<0.001
TNFα, pg mL^−1^	53.01 ± 1.99	49.11 ± 2.38	48.74 ± 1.76	52.68 ± 2.56	0.224
GSH-PX, U mL^−1^	580.23 ± 38.00 ^a^	646.27 ± 23.30 ^a,b^	683.44 ± 36.46 ^b^	604.81 ± 17.05 ^a,b^	0.027
OH U mL^−1^	603.18 ± 10.77 ^a^	607.34 ± 24.54 ^a^	661.34 ± 8.32 ^b^	612.88 ± 18.44 ^a,b^	0.034
SOD U mL^−1^	19.30 ± 2.34	26.17 ± 2.19	22.29 ± 1.76	23.43 ± 3.81	0.08
T-AOC mMol L^−1^	0.76 ± 0.02 ^a,b^	0.72 ± 0.02 ^a^	0.77 ± 0.01 ^a,b^	0.79 ± 0.02 ^b^	0.034
H_2_O_2_ mMol L^−1^	74.66 ± 6.50	64.67 ± 4.33	63.72 ± 3.92	66.11 ± 4.89	0.171
NO µmol mL^−1^	77.14 ± 7.94 ^a^	124.28 ± 7.40 ^c^	101.90 ± 5.60 ^b^	97.62 ± 6.41 ^b^	0.001
ALT ^3^ U L^−1^	74.12 ± 1.22 ^a^	79.98 ± 2.43 ^b^	75.97 ± 1.44 ^a,b^	75.61 ± 1.90 ^a,b^	0.033
AST ^3^ U L^−1^	33.14 ± 0.94 ^b^	34.68 ± 1.44 ^b^	29.35 ± 0.80 ^c^	43.45 ± 1.55 ^a^	<0.001
ALP ^3^ U L^−1^	45.11 ± 1.68	44.93 ± 1.56	42.43 ± 1.58	43.97 ± 1.29	0.273
γ-GT ^3^ U L^−1^	28.12 ± 1.16 ^a^	25.87 ± 1.21 ^a^	26.61 ± 1.09 ^a^	32.98 ± 1.19 ^b^	0.002
CHE ^3^ U L^−1^	460.86 ± 12.31 ^a^	756.90 ± 8.94 ^c^	561.46 ± 14.27 ^b^	741.74 ± 17.50 ^c^	<0.001

^a–c^ Different superscripts within a row indicate a significant difference (*p* < 0.05). ^1^ Dietary treatments: CON = basal diet; AGP = basal diet supplemented with 0.075 g/kg chlortetracycline, 0.055 g/kg kitasamycin, and 0.01 g/kg virginiamycin; CBN = basal diet supplemented with 2.5 × 10^8^ CFU/kg *C. butyricum*; CBH = basal diet supplemented with 2.5 × 10^9^ CFU/kg *C. butyricum*. ^2^ SE: standard error of the mean. Each treatment group contains six replicates (*n* = 6), and each replicate includes one piglet. ^3^ IgA immunoglobulin A; IgM immunoglobulin M; IgG immunoglobulin G; IL-1β, interleukin 1β; IL-2, interleukin 2; IL-6, interleukin 6; TNF-α, tumor necrosis factor α; GSH-PX, glutathione peroxidase; OH, inhibiting ability of hydroxyl radical; SOD, superoxide dismutase; T-AOC, total antioxidant capacity; H_2_O_2_, hydrogen peroxide; NO, nitric oxide; ALT, alanine transaminase; AST, aspartate aminotransferase; ALP, alkaline phosphatase; γ-GT, gamma-glutamyl transpeptidase; CHE, Cholinesterase.

**Table 6:** Effect of dietary *C. butyricum* on the intestinal morphology of piglets (mean ± SE ^2^).

Item	Dietary Treatments ^1^				*p*-Value
	CON	AGP	CBN	CBH	
Duodenum					
Villus height, μm	651.99 ± 13.56	670.89 ± 8.61	656.67 ± 9.29	680.22 ± 19.32	0.183
Crypt depth, μm	288.83 ± 9.96	293.41 ± 8.42	299.29 ± 6.82	304.47 ± 10.00	0.266
Villus height/Crypt depth	2.27 ± 0.05	2.30 ± 0.06	2.20 ± 0.05	2.25 ± 0.07	0.309
Jejunum					
Villus height, μm	658.99 ± 19.02 ^a^	695.39 ± 14.33 ^a,b^	694.36 ± 15.66 ^a,b^	727.42 ± 11.10 ^b^	0.030
Crypt depth, μm	276.35 ± 6.32 ^a^	309.53 ± 13.10 ^a,b^	300.30 ± 15.01 ^a,b^	316.41 ± 11.11 ^b^	0.022
Villus height/Crypt depth	2.38 ± 0.06	2.27 ± 0.09	2.35 ± 0.09	2.33 ± 0.09	0.393
Ileum					
Villus height, μm	568.06 ± 12.61	584.11 ± 22.18	602.39 ± 25.57	605.67 ± 23.70	0.270
Crypt depth, μm	251.42 ± 9.07	245.86 ± 8.80	261.72 ± 14.49	252.95 ± 9.01	0.344
Villus height/Crypt depth	2.28 ± 0.08	2.38 ± 0.08	2.35 ± 0.12	2.40 ± 0.07	0.400

^a,b^ Different superscripts within a row indicate a significant difference (*p* < 0.05). ^1^ Dietary treatments: CON = basal diet; AGP = basal diet supplemented with 0.075 g/kg chlortetracycline, 0.055 g/kg kitasamycin, and 0.01 g/kg virginiamycin; CBN = basal diet supplemented with 2.5 × 10^8^ CFU/kg *C. butyricum*; CBH = basal diet supplemented with 2.5 × 10^9^ CFU/kg *C. butyricum*.^2^ SE: standard error of the mean. Each treatment group contains six replicates (*n* = 6), and each replicate includes one male piglet.

**Table 7:** Effect of dietary *C. butyricum* on the relative abundance (%) of microbiota in the colonic chyme of piglets (mean ± SE ^2^).

Phyla/Genera	Dietary Treatments ^1^				*p*-Value
	CON	AGP	CBN	CBH	
*Firmicutes* (phylum)	63.67 ± 7.82	66.06 ± 2.52	57.15 ± 3.86	69.07 ± 5.37	0.158
*Sarcina*	8.61 ± 3.34	3.86 ± 1.28	3.70 ± 0.85	4.89 ± 1.71	0.131
*Subdoligranulum*	5.72 ± 2.58	2.54 ± 0.88	2.24 ± 0.23	2.55 ± 0.82	0.129
*Streptococcus*	4.52 ± 1.25 ^a^	7.81 ± 1.84 ^a,b^	8.51 ± 2.26 ^a,b^	11.46 ± 2.34 ^b^	0.022
*Blautia*	6.20 ± 1.93	5.97 ± 1.30	3.67 ± 1.32	6.73 ± 2.42	0.286
*Butyricicoccus*	2.01 ± 0.67	1.33 ± 0.25	2.48 ± 0.82	2.24 ± 0.70	0.266
*Megamonas*	0.10 ± 0.07	0.79 ± 0.75	0.007 ± 0.002	0.02 ± 0.01	0.189
*Phascolarctobacterium*	1.25 ± 0.32	0.92 ± 0.21	1.62 ± 0.39	1.46 ± 0.60	0.279
*Mitsuokella*	0.22 ± 0.08	0.60 ± 0.57	0.17 ± 0.09	0.05 ± 0.02	0.238
*Anaerostipes*	0.12 ± 0.08	1.15 ± 0.17	0.92 ± 0.16	1.75 ± 0.49	0.180
*Roseburia*	1.80 ± 0.50	1.04 ± 0.32	1.01 ± 0.57	1.26 ± 0.40	0.190
*Faecalibacterium*	0.69 ± 0.23	0.94 ± 0.17	0.40 ± 0.07	0.77 ± 0.42	0.188
*Peptococcus*	0.33 ± 0.09	0.41 ± 0.08	0.54 ± 0.12	0.40 ± 0.07	0.175
*Turicibacter*	0.01 ± 0.006	0.008 ± 0.004	0.05 ± 0.03	0.26 ± 0.17	0.078
*Proteobacteria* (phylum)	14.95 ± 8.72	7.98 ± 1.90	6.76 ± 1.74	6.56 ± 2.28	0.248
*Actinobacillus*	2.71 ± 1.45	2.44 ± 1.14	0.96 ± 0.29	2.62 ± 0.58	0.261
*Bacteroidetes* (phylum)	14.73 ± 4.20	19.60 ± 4.03	19.40 ± 3.76	15.24 ± 4.13	0.445
*Alloprevotella*	2.33 ± 1.18	0.60 ± 0.14	1.90 ± 0.91	0.74 ± 0.37	0.163
*Parabacteroides*	0.20 ± 0.03	0.80 ± 0.48	0.38 ± 0.13	0.35 ± 0.18	0.154
*Unidentified Bacteroidales*	0.48 ± 0.26	0.26 ± 0.16	0.76 ± 0.42	0.09 ± 0.03	0.116
*Actinobacteria* (phylum)	0.61 ± 0.14	0.70 ± 0.14	1.11 ± 0.31	0.56 ±0.02	0.072
*Bifidobacterium*	0.03 ± 0.01 ^a^	0.04 ± 0.02 ^a^	0.25 ± 0.12 ^b^	0.04 ± 0.01 ^a^	0.039

^a,b^ Different superscripts within a row indicate a significant difference (*p* < 0.05). ^1^ Dietary treatments: CON = basal diet; AGP = basal diet supplemented with 0.075 g/kg chlortetracycline, 0.055 g/kg kitasamycin, and 0.01 g/kg virginiamycin; CBN = basal diet supplemented with 2.5 × 10^8^ CFU/kg *C. butyricum*; CBH = basal diet supplemented with 2.5 × 10^9^ CFU/kg *C. butyricum*. ^2^ SE: standard error of the mean. Each treatment group contains six replicates (*n* = 6), and each replicate includes one male piglet.
